# Joint-Preserving Surgeries for Hallux Rigidus Based on Etiology: A Review and Commentary

**DOI:** 10.3390/jcm14051595

**Published:** 2025-02-26

**Authors:** Kenichiro Nakajima

**Affiliations:** Center for Foot and Ankle Surgery, Department of Orthopedic Surgery, Yashio Central General Hospital, Yashio 340-0814, Saitama, Japan; nakajimakenichiro@hotmail.co.jp

**Keywords:** hallux rigidus, hallux limitus, metatarsophalangeal joint, etiology, osteotomy, arthroscopy

## Abstract

In 1927, Cochrane noted that elastic resistance to dorsiflexion of the hallux was retained after the cheilectomy or dorsiflexion osteotomy of the metatarsal head and speculated that the shortening and tightness of the soft tissues below the first metatarsophalangeal joint would be the etiology of hallux rigidus. He devised a novel surgery in which the plantar tissues were divided using a plantar approach and reported good results in 12 patients with the disappearance of elastic resistance during dorsiflexion and with no recurrence. Although he identified the etiology of hallux rigidus and developed a revolutionary surgery that directly addressed the etiology, this approach has not yet been seen in current surgeries. Therefore, we hypothesized that current surgeries for hallux rigidus lack rationality regarding etiology and aimed to critically review joint-preserving surgeries based on etiology. First, we summarized the literature on proposed causes and explained how the condition progresses from shortened, tightened plantar soft tissues. We then reviewed joint-preserving surgeries in terms of etiology and treatment efficacy and finally mentioned the arthroscopic Cochrane procedure as a promising option.

## 1. Introduction—The Etiology of Hallux Rigidus

In 1927, Cochrane [1] noted that the elastic resistance in hallux dorsiflexion remained after the cheilectomy or dorsiflexion osteotomy of the metatarsal head. He hypothesized that this hallux rigidus was attributed to shortened and contracted structures on the plantar aspect of the joint. To address this, a novel surgery was devised, wherein a longitudinal skin incision was made over the plantar aspect of the first metatarsophalangeal (MTP) joint, the flexor hallucis longus (FHL) tendon was secured, and the joint capsule, flexor hallucis brevis (FHB) tendon, and plantar portion of the lateral ligament were divided. This surgery was performed on 12 patients with hallux rigidus, and all were satisfied with the outcomes. Notably, the elastic resistance during dorsiflexion disappeared, and there were no cases of recurrence. However, despite this great breakthrough, such an approach has not yet been seen in current surgeries.

Therefore, we hypothesized that current surgeries for hallux rigidus lack rationality regarding etiology and aimed to critically review joint-preserving surgeries based on etiology. First, we summarized the literature on proposed causes and explained how the condition progresses from shortened, tightened plantar soft tissues. We then reviewed joint-preserving surgeries in terms of etiology and treatment efficacy and finally mentioned the arthroscopic Cochrane procedure as a promising option.

## 2. The Pathological Changes in Hallux Rigidus

### 2.1. Dorsal Impingement

**Figure 1:**
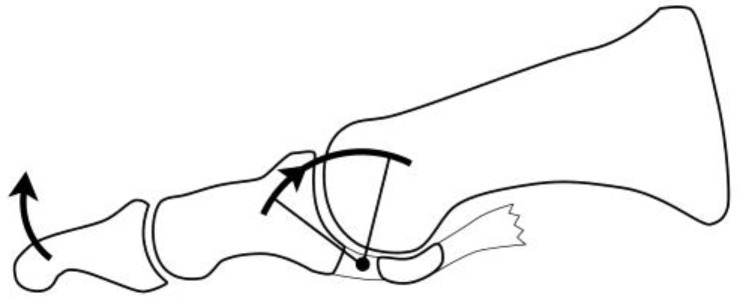
Dorsal impingement. When the sesamoid bones are immobile, the proximal phalanx moves like a windshield wiper around the base of the MTP joint. Consequently, the metatarsal head is impinged by the dorsal margin of the proximal phalanx. (This figure is modified from Camasta et al., *Clin Podiatr Med Surg* 1996 [2]).

As illustrated by Camasta [2] (Figure 1), when the soft tissues at the bottom of the first MTP joint in the hallux rigidus contract, the proximal phalanx of the hallux undergoes wire motion centered on that area, similar to a windshield wiper. This causes impingement between the metatarsal head and the dorsal side of the proximal phalanx.

Dorsal impingement causes cartilage defects on the dorsal metatarsal head. Bingold and Collins [3] performed a pathological examination of the surgically resected metatarsal head, which revealed the loss of superficial cartilage layers and irregularity of the calcified line at the base of the articular cartilage. It was hypothesized that FHB contracture causes increased compressive force on the dorsal articular surface, resulting in arthritic changes. Similarly, McMaster [4] showed a pathological photograph of the separating flap of the dorsal articular cartilage of the metatarsal head in a patient with hallux rigidus. It was suspected that the defect caused impingement of the proximal phalanx edge on the dorsal metatarsal head. A previous study by Nakajima [5] on MTP joint arthroscopy included photographs of a case of hallux rigidus, wherein the articular cartilage was lost from the center to the dorsal third of the articular surface, but the cartilage was preserved above that and at the dorsal spur. 

Dorsal impingement of the hallux rigidus has also been studied using kinematic approaches. Sheff et al. [6] investigated the axis of motion of an articular surface in cadaveric X-rays. Under normal conditions, the proximal phalanx glided on the articular surface of the metatarsal bone. In hallux rigidus, there was decreased joint movement between the sesamoid bones and the metatarsal head, and the proximal phalanx surface appeared to be jammed against the metatarsal surface. Flavin et al. [7] found that the stress of the articular cartilage on the dorsal third of the metatarsal head increased in the presence of contractures, from 3.6 MPa under normal loading to 4.3 MPa under a 30% increase in the FHB contracture and to 7.3 MPa under a 30% increase in the plantar fascia contracture. Therefore, contractures of these tissues can damage the articular cartilage on the dorsal third of the metatarsal head.

### 2.2. Windlass Mechanism and Functional Hallux Rigidus 

**Figure 2:**
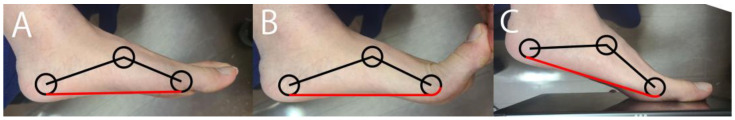
Windlass mechanism and hallux rigidus. (**A**) The longitudinal arch of the foot in the non-weight-bearing position. (**B**) Dorsiflexion in the non-weight-bearing position: the longitudinal arch heightens, and the plantar fascia rolls up around the hallux metatarsophalangeal (MTP) joint. (**C**) Dorsiflexion while weight-bearing: the plantar fascia is stretched, which restricts the heightening of the longitudinal arch and rolling of the plantar fascia, thereby limiting dorsiflexion. This tendency was evident in hallux rigidus because of the intrinsic tightness or shortening of the plantar fascia.

The windlass mechanism explains the restriction of dorsiflexion under the weight-bearing conditions of a normal foot [8] (Figure 2). Under non-weight-bearing conditions, the longitudinal arch heightens as the hallux dorsiflexes, and the plantar fascia rolls up to the first MTP joint. Meanwhile, under weight-bearing conditions where the plantar fascia is stretched, the heightening of the longitudinal arch and rolling-up of the plantar fascia are both restricted, thereby limiting dorsiflexion.

**Figure 3:**
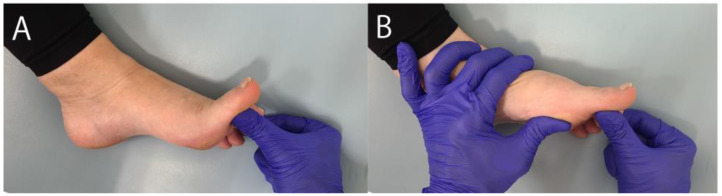
Functional hallux rigidus test. (**A**) Test simulating non-weight-bearing. The hallux is passively dorsiflexed without pressing the metatarsal head. (**B**) Test simulating weight-bearing. The hallux is passively dorsiflexed while pushing the metatarsal head upward from below. Functional hallux rigidus is indicated by dorsiflexion angles of 65–90° in the non-weight-bearing test and <0° in the weight-bearing test.

This tendency was especially evident in the hallux rigidus due to the slight tightness or shortening of the plantar soft tissues. Dananberg [9,10] coined the term “functional hallux rigidus” to describe the condition wherein dorsiflexion is noticeably restricted only during weight-bearing but not otherwise. A review article by Maceira and Monteagudo [11] demonstrated a manual test for functional hallux rigidus (Figure 3), wherein functional hallux rigidus is indicated by dorsiflexion angles of 65–90° during the non-weight-bearing test and <0° during the weight-bearing test. Nevertheless, the shortening and contracture of plantar soft tissues can be restored in functional hallux rigidus. Shrmus et al. [12] designed a comparative experimental study including 20 patients with functional hallux rigidus. After 1 month of sesamoid mobilizations, flexor hallucis strengthening exercises, and gait training, the experimental group achieved a greater hallux dorsiflexion angle and less pain versus the control group.

### 2.3. Structural Change from Functional Hallux Rigidus to Metatarsal Primus Elevatus 

**Figure 4:**
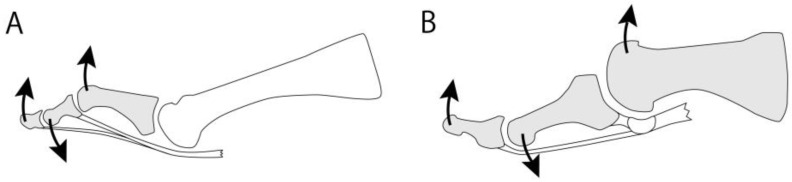
Similarity between hallux rigidus and hammer toe. (**A**) Hammer toe. (**B**) Hallux rigidus.

Camasta [2] discussed the similarity of bone alignment between hallux rigidus and hammer toe (Figure 4). When the tendon is shortened, the most distal bone touches the ground, the second most distal bone plantarflexes, and the third most distal bone dorsiflexes during weight-bearing to stabilize the toe. In this situation, the proximal phalanx is elevated in the hammer toe, while the metatarsal is elevated in the hallux rigidus.

The structural change from functional hallux rigidus to hallux primus elevatus in hallux rigidus has been supported by the literature. Horton et al. [13] compared the weight-bearing lateral radiographs of 81 patients with hallux rigidus and 50 normal controls. The mean metatarsal elevation of patients with mild or moderate hallux rigidus was comparable to that of controls, whereas this was slightly higher among patients with advanced hallux rigidus versus controls. Similarly, Coughlin et al. [14] found no metatarsal elevation in grades 1 and 2 hallux rigidus, but metatarsal elevation was more common in grades 3 and 4 hallux rigidus versus controls. Furthermore, Shurnas [15] proposed that metatarsal elevation occurs secondary to arthritic changes and decreased range of motion of the MTP joint. In contrast, studies that did not differentiate between the stages of hallux rigidus had conflicting results regarding metatarsal elevation. In a study of weight-bearing radiographs, Meyer et al. [16] found that the average elevation of the first metatarsal was 6.91 mm in 120 randomly selected feet and 5.95 mm in 22 feet with hallux rigidus. This finding cast doubt regarding the existence of the hallux primus elevatus in hallux rigidus. Meanwhile, Roukis [17] compared 100 cases of hallux rigidus versus patients with hallux valgus, plantar fasciitis, and Morton’s disease, revealing a significant elevation of the first metatarsal with respect to the second metatarsal in the hallux rigidus group. Jones et al. [18] devised a method for measuring metatarsal elevation in relation to the proximal phalanx and reported that the first metatarsal was significantly elevated in hallux rigidus versus controls, with high intra- and inter-rater evaluations. Additionally, Anwandar et al. [19] found significant metatarsal elevation when comparing 25 patients with hallux rigidus versus 50 healthy individuals, with an intraclass correlation coefficient of 0.93 between both observers. These findings suggest that differentiating between the stages of hallux rigidus is essential when evaluating hallux primus elevatus.

### 2.4. Long Metatarsal

The long metatarsal is a controversial etiology of hallux rigidus. To the best of our knowledge, studies discussing the metatarsal length in hallux rigidus are scarce. Among the existing studies, Nilsonne [20] evaluated the length of the first metatarsal in 497 normal feet and 49 feet with hallux rigidus. Using the tip of the second metatarsal as a reference, a positive index, zero index, and negative index, respectively, were observed in 34%, 13%, and 52% of normal feet and in 81%, 14%, and 5% of feet with hallux rigidus. However, a comparison of radiographs of patients with and without (*n* = 51 each) hallux rigidus by Zgonis et al. [21] revealed significantly shorter metatarsal length in patients with hallux rigidus (65.4 vs. 67.7 mm), thus casting doubt on its role in the etiology of hallux rigidus. Meanwhile, in a study of 132 cases with hallux rigidus and a control group of 132 normal feet, Calvo et al. [22] found that the ratio of the metatarsal length to the total foot length was significantly greater in hallux rigidus versus controls. These findings suggest that having a long first metatarsal does not cause limited dorsiflexion unless the tissue on the plantar aspect of the first MTP joint restricts dorsiflexion. However, a long first metatarsal may tend to shorten the plantar tissues. 

## 3. Joint-Preserving Surgeries for Hallux Rigidus Based on Etiology

### 3.1. Metatarsal Decompression Osteotomy

Due to the shape of the osteotomy, metatarsal decompression osteotomy is used in the surgical management of hallux primus elevatus and long metatarsals, although its application for each grade varies [23,24,25,26,27,28,29,30]. However, Derner et al. [31] stated that metatarsal decompression osteotomy can alleviate pain in hallux rigidus by reducing the compressive force on the dorsal metatarsal head and loosening the soft tissues below the joint. 

**Figure 5:**
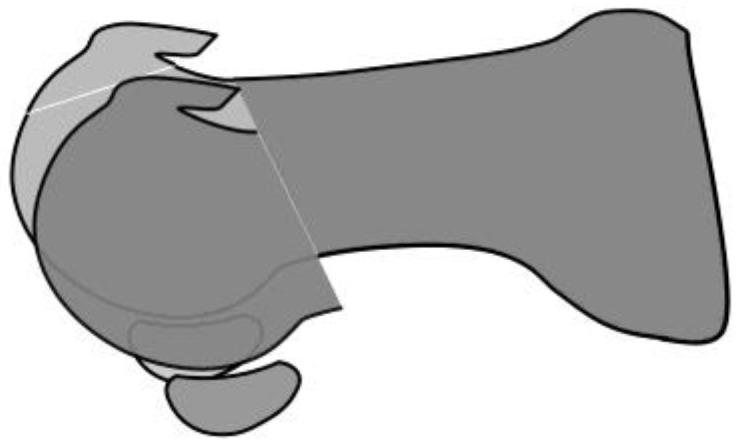
Metatarsal decompression osteotomy and cheilectomy. Metatarsal decompression osteotomy (dark gray) shifts the metatarsal head proximally and toward the plantar side, which reduces compressive pressure on the dorsal third of the metatarsal bone. Cheilectomy (light gray) alleviates pressure on this area by resecting the dorsal third of the metatarsal head. Meanwhile, removing the dorsal spur is not necessary in metatarsal decompression osteotomy because it already achieves decompression by lowering the metatarsal head. Both procedures are similar in terms of reducing the compressive pressure on the dorsal third of the metatarsal head, but they differ in the position of the sesamoids. In metatarsal decompression osteotomy, the metatarsal head and sesamoid bones are shifted proximally, thereby loosening the tight plantar tissues. In cheilectomy, the tension of the plantar tissues remains unchanged because the positions of the metatarsal head and sesamoids remain unchanged, eventually leading to arthritic changes.

Decompression osteotomy includes various methods such as Youngswick osteotomy [26,27,28,29,30,32] and oblique osteotomy [23,24,25,33,34,35], which usually result in favorable outcomes except after modified Reverdin Green osteotomy [23,24,25,26,27,28,29,30,31,33,34,35,36]. Only one comparative study has been performed between osteotomies, which reported similar outcomes between Youngswick and oblique osteotomies [37]. However, because the metatarsal osteotomies reported so far were combined with cheilectomies, it remains unclear whether the improved outcomes were associated with metatarsal osteotomies or cheilectomies [38]. Nakajima [33] believes that cheilectomy is unnecessary if metatarsal osteotomy is able to reduce the compressive force on the dorsal metatarsal head, reporting good outcomes after metatarsal osteotomy without cheilectomy (Figure 5).

Recent studies have reported the effectiveness of metatarsal decompression osteotomy across all grades of hallux rigidus. Saur et al. [34] reported that the American Orthopedic Foot & Ankle Society (AOFAS) scores improved from 54 to 92 in 87 feet across all stages of hallux rigidus after a mean follow-up of 51 months, with scores of 95% of patients being excellent or good. Two studies by Nakajima [33,35] also reported the effectivity of metatarsal decompression osteotomy in 119 feet across all grades, all with similar improvements in pain. This suggests that plantar soft tissue contracture and dorsal impingement occur in all grades of hallux rigidus, which can be effectively relieved by metatarsal osteotomy in all grades.

One concern in metatarsal decompression osteotomy is plantar pain due to the plantar shift in the metatarsal head. Zammit et al. [39] reported that plantar pressure of hallux rigidus feet was lower under the first MTP joint compared to the first distal phalanx and the second to fourth MTP joints. Bryant et al. [40] reported that plantar pressure under the first MTP joint did not increase after Youngswick osteotomy (n = 17) compared to controls (n = 23). Meanwhile, Nakajima reported that 28% of patients felt discomfort under the metatarsal head after a 7 mm plantar shift in the metatarsal head [33], whereas only 5% felt discomfort after a 3 mm plantar shift [35].

### 3.2. Metatarsal Dorsiflexion Osteotomy

**Figure 6:**
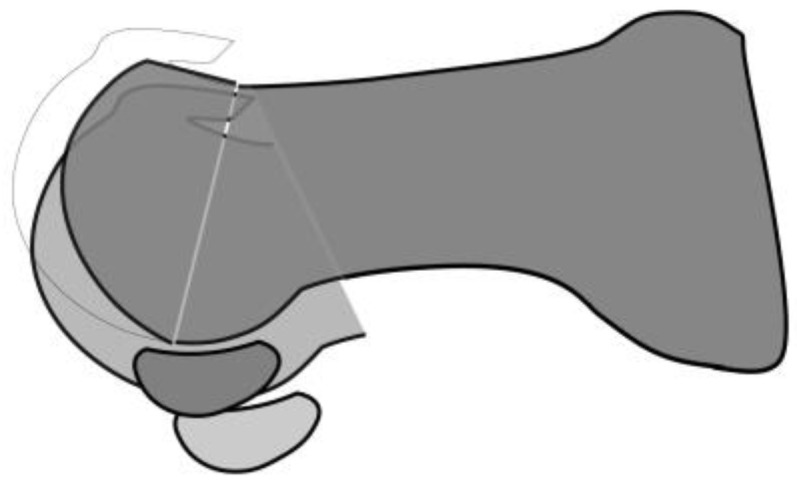
Decompression and dorsiflexion osteotomy. In dorsiflexion osteotomy (dark gray), the metatarsal head is higher, and the sesamoid bones are located more distally compared to decompression osteotomy (light gray). Dorsiflexion osteotomy does not sufficiently loosen the soft tissue on the plantar side or reduce compressive pressure on the metatarsal head.

Metatarsal dorsiflexion osteotomy appears to have a similar effect in reducing compressive force on the dorsal articular surface. However, recent studies have reported poor outcomes of dorsiflexion osteotomy in high-grade hallux rigidus [41,42]. Although both osteotomies reduce compressive force on the dorsal third of the first MTP joint surface, decompression osteotomy loosens the soft tissues below the joint due to the plantar shift in the metatarsal head, whereas dorsiflexion osteotomy does not (Figure 6). Since dorsiflexion osteotomy does not sufficiently alleviate the compressive force on the joint surface, it results in poor pain relief.

### 3.3. Cheilectomy

Cheilectomy is the gold standard for early-stage hallux rigidus [43,44], but some recent studies have reported poor results [45,46,47,48,49]. Hattrup and Johnson [45] reviewed 58 patients who underwent cheilectomy, reporting dissatisfaction rates of 15%, 31.8%, and 37.5% among patients with grade 1, 2, and 3 hallux rigidus, respectively. Harrison et al. [46] evaluated 25 patients who underwent cheilectomy with a mean follow-up of 17 months using the Manchester-Oxford Foot and Ankle Questionnaire (MOXFQ); only 84% of patients showed improvements in gait, 68% in the social domain, and 59% in the pain domain, while 3 patients subsequently required arthrodesis. A systematic review by Roukis et al. [47] reported that among 706 isolated cheilectomies for hallux rigidus, 8.8% underwent surgical revision of arthrodesis. Sidon et al. [48] found that among 165 patients (169 feet) who underwent cheilectomy with an average follow-up of 6.6 years, 31% felt neither satisfied nor dissatisfied, dissatisfied, or very dissatisfied, 5.3% underwent reoperation, and 24.9% would prefer not to repeat the surgery in the same situation. Lastly, Teoh et al. [49] evaluated 89 patients (98 feet) who underwent cheilectomy with a mean follow-up of 50 months, revealing improvements in visual analog scale (VAS) score (80 to 30) and MOXFQ score (58.6 to 30.5). Additionally, 12% underwent reoperation, and 7% underwent arthrodesis.

Several studies have reported that arthritic changes progress after cheilectomy. Mulier et al. [50] performed cheilectomy in 20 athletes (22 feet) with an average age of 31 years with grade 1 or 2 hallux rigidus and reported fair, good, and excellent ratings in 1, 7, and 14 patients, respectively, using their own clinical score sheet, but on follow-up, 7 out of 13 patients had progression of radiographic arthritic changes. Easley et al. [51] performed cheilectomy in 57 patients (75 feet) with an average follow-up of 63 months, revealing improvements in AOFAS score (45 to 85) and dorsiflexion angle (34° to 64°). However, a considerable number progressed from grade 1 to grades 2 and 3 (9/17 and 6/17, respectively) and from grade 2 to grade 3 (24/39). A review by Tomilinson [52] reported that conversion from cheilectomy to arthrodesis was necessary for up to 25% of patients. This may be because cheilectomy does not move the hallux sesamoids proximally, causing the plantar soft tissue to remain tight and the pressure on the femoral head to persist (Figure 5). 

Although cheilectomy is essentially a joint-destructive surgery, it is still performed in patients with early-stage hallux rigidus. Mann and Clanton [53] demonstrated a cheilectomy that removes the dorsal fourth to third of the articular surface, similar to that of Coughlin et al. [54]. Because of its destructive nature, normal joint movement is lost. A cadaveric study by Heller et al. [55] reported that cheilectomy increased the dorsiflexion angle but replaced the normal sliding movement with a pivot shift movement. 

To the best of our knowledge, there has been only one comparative study between cheilectomy and metatarsal decompression osteotomy. In this study, Cullen et al. [56] compared cheilectomy (341 feet) versus decompression osteotomy (82 feet), revealing a significantly higher 5-year reoperation rate for cheilectomy (8.2% vs. 1.2%).

Cheilectomy is a form of symptomatic treatment that removes the dorsal articular surface where impingement pain occurs, but this does not address the underlying etiology. Thus, Cochrane [1] regarded cheilectomy as illogical. Meanwhile, Geldwert et al. [57] argue that cheilectomy without addressing the underlying etiology is a disadvantage, although they reported good outcomes. Cheilectomy is essential, especially for treating intractable keratosis above the dorsal spur. However, given the abovementioned issues, its status as the gold standard for early-stage hallux rigidus is questionable.

### 3.4. Arthroscopic Surgeries

#### 3.4.1. Arthroscopic Cheilectomy

**Figure 7:**
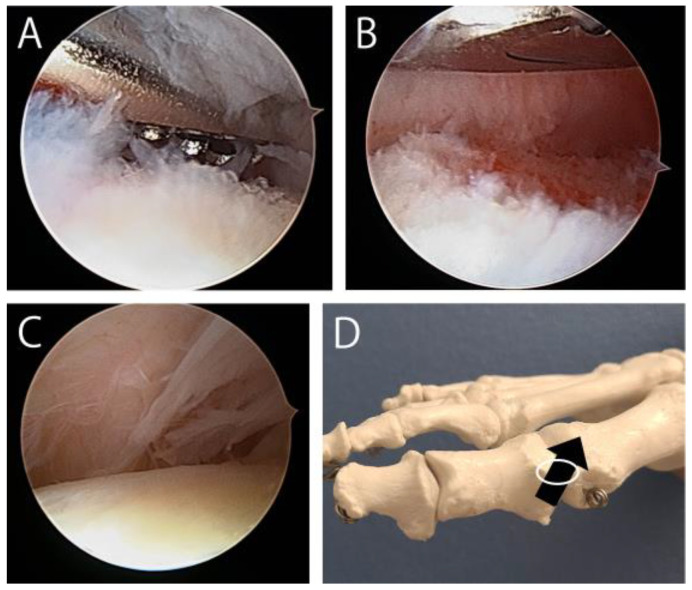
Images of arthroscopic cheilectomy. (**A**) Arthroscopic resection of the dorsal spur. A bone spur (white tissue at the bottom of the image) occupies the dorsal first MTP joint space. This results in a narrow joint space, making it difficult to determine the shape of the spur. (**B**) The surgical site after the spur resection. A shaver was used to lift the joint capsule and broaden the arthroscopic view. Because the site is narrow, it is difficult to confirm whether sufficient spur resection has been achieved. (**C**) Normal arthroscopic view of the dorsal MTP joint space in a patient with hallux sesamoid disorder. There is sufficient space between the metatarsal head and the joint capsule. (**D**) Photograph indicating the dorsomedial portal (circle) and the arthroscope direction (arrow).

An increasing number of studies have investigated arthroscopic cheilectomy. Some have reported that dorsal exostosis can be resected under arthroscopic guidance [58,59,60,61,62], but none have provided photographs of this process. Therefore, the specifics of how the exostosis is viewed and removed under arthroscopic guidance remain unclear. Arthroscopically resecting the exostosis can be difficult because of the following reasons. First, since the exostosis occupies the dorsal first MTP joint space, it can be difficult to effectively manipulate both an arthroscope and an abrader in this narrow space [5]. Second, it can be difficult to determine the entire shape of the exostosis during arthroscopic resection. Third, surgeons who are unfamiliar with the first MTP joint arthroscopy can have difficulty in differentiating between the normal dorsal view versus the presence of an exostosis (Figure 7). To overcome these issues, several recent reports introduced a technique wherein the exostosis was first removed under fluoroscopy; then, the joint space was arthroscopically observed [63,64,65,66,67]. Another report used ultrasound instead of fluoroscopy [68].

Similar clinical outcomes have been reported for arthroscopic and open cheilectomies. Hickey et al. [63] performed arthroscopic cheilectomy in 36 patients with a mean follow-up of 4.7 years, revealing that 28% had no pain, 56% had mild pain (VAS score 34), and 1 patient had extensor hallucis longus tendon rupture. Furthermore, 83% of the patients reported that they would recommend this surgery. Moreover, in a study of 20 patients who underwent arthroscopic cheilectomy, Glenn et al. [64] reported improvements in VAS score (70.5 to 7.5) and dorsiflexion angle (32° to 48°) after 16.5 months. Additionally, Di Nallo et al. [67] conducted a retrospective multicenter cohort study among 28 patients (30 feet) who underwent arthroscopic cheilectomy. After an average follow-up of 4 years, 97% of patients were satisfied, with notable improvements in dorsiflexion angle (42° to 46°) and AOFAS score (59 to 84). These results are similar to those of open cheilectomy, and simply reducing the wound size does not necessarily lead to better treatment outcomes.

#### 3.4.2. Arthroscopic Cochrane Procedure

Considering the aforementioned issues on cheilectomy, arthroscopy in hallux rigidus would be better suited for observing and treating the plantar side of the joint rather than for cheilectomy. For example, arthroscopic observation and treatment of the tightness around the sesamoid bones can help elucidate the pathology of the hallux rigidus. Furthermore, the arthroscopic Cochrane procedure holds potential, although only one technical note has been published regarding this procedure without reporting clinical outcomes [69]. The arthroscopic Cochrane procedure may be applicable to all stages of hallux rigidus, as it directly addresses shortened, tightened plantar soft tissues. Partial release may suffice for early stages, whereas complete release is necessary for late stages. One concern regarding the Cochrane procedure is the postoperative risk of developing a cock-up deformity due to the division of the FHB tendon. However, the development of a postoperative hallux deformity is unlikely due to the degenerative changes and limited range of motion of the first MTP joint [69,70].

In summary, Table 1 lists joint-preserving surgeries for hallux rigidus, addressing plantar soft tissue shortening, dorsal impingement, advantages, disadvantages, major complications, and their applicability to hallux rigidus stages based on Coughlin’s classification [43].

## 4. Conclusions

Cochrane identified that the shortening and tightness of the plantar soft tissues below the first MTP joint is the etiology of hallux rigidus. Plantar soft tissue shortening and tightness cause dorsal impingement at the metatarsal head. When the plantar soft tissues shorten, functional hallux rigidus occurs first, followed by metatarsal elevation.

Metatarsal decompression osteotomy addresses plantar soft tissue shortening, tightness, metatarsal elevation, and dorsal impingement by shifting the metatarsal head plantarward and proximally. It has shown good outcomes for all grades of hallux rigidus.

Metatarsal head dorsiflexion osteotomy addresses dorsal impingement by dorsiflexing the metatarsal head but does not correct plantar soft tissue shortening and tightness, which may lead to poor outcomes in the late stage of hallux rigidus.

Cheilectomy is the current gold standard for early-stage hallux rigidus. It addresses dorsal impingement by resecting the dorsal third of the metatarsal head but does not correct plantar soft tissue shortening and tightness, which may increase the risk of arthritic progression and reoperation. Additionally, resecting the dorsal third of the metatarsal head replaces the normal sliding joint movement with a pivot shift movement. Considering this, it is questionable whether this procedure should remain the gold standard for the early stages of hallux rigidus.

Recently, arthroscopic cheilectomy reports have increased, but considering the issues of cheilectomy, the use of arthroscopy for hallux rigidus may be more meaningful for elucidating its intra-articular pathogenesis and for arthroscopic Cochran’s surgery.



This is a narrative review, not a systematic one. Therefore, references were not collected or cited under strict inclusion and exclusion criteria. PubMed was used to search for references using the terms “hallux rigidus”, “etiology”, “pathology”, “functional hallux rigidus”, “osteotomy”, “surgery”, “cheilectomy”, and “arthroscopy”. Titles and abstracts of candidate papers were screened, and those deemed suitable were collected and stored in Mendeley. Relevant papers were cited appropriately. Although arbitrary citation was avoided, studies reporting poor outcomes for cheilectomy were cited for critical review. A study on hallux sesamoid shape was excluded from the pathology references due to unclear sesamoid length measurements.

## Figures and Tables

**Table 1:** Joint-preserving surgeries for hallux rigidus based on the etiology.

Surgical Option	How to Address the Shortening Plantar Tissues	How to Address Dorsal Impingement	Advantages	Disadvantages and Major Complications	Possible Applications to Hallux Rigidus Stages
Metatarsal decompression osteotomy	By shifting the metatarsal head plantarward and proximally	By shifting the metatarsal head plantar and proximal	Preserve articular surface Simple procedure Lower reoperation rate than cheilectomy	Plantar discomfort or pain	Stages 1, 2, 3, and 4
Metatarsal head dorsiflexion osteotomy	Not address	By dorsiflexing the metatarsal head	Preserve articular surface	Poor outcomes in severe hallux rigidus	Stage 1, 2
Cheilectomy	Not address	By resecting the dorsal third metatarsal head	Treat keratosis above the dorsal spur	Partially Sacrificing the metatarsal head Arthritic change progression Pivot shift joint movement	For keratosis above the dorsal spur Stage 1, 2
Arthroscopic Cochrane procedure	By directly releasing the plantar soft tissues	By directly releasing the plantar soft tissues	Minimally invasive Preserve articular surface Directly address the etiology	Cock-up deformity?	Partial release for stages 1, 2 Complete release for stages 3, 4

## Abbreviations

The following abbreviations are used in this manuscript:

AOFAS	American Orthopedic Foot & Ankle Society
FHB	Flexor hallucis brevis
FHL	Flexor hallucis longus
MOXFQ	Manchester–Oxford Foot and Ankle Questionnaire
MTP	Metatarsophalangeal
VAS	Visual analog scale
